# Assessment of Fungal Contamination in Fish Feed from the Lake Victoria Basin, Uganda

**DOI:** 10.3390/toxins12040233

**Published:** 2020-04-07

**Authors:** Victoria Tibenda Namulawa, Samuel Mutiga, Fred Musimbi, Sundy Akello, Fredrick Ngángá, Leah Kago, Martina Kyallo, Jagger Harvey, Sita Ghimire

**Affiliations:** 1 National Agricultural Research Organization, Aquaculture Research & Development Center, P.O. Box 530, Kampala 00256, Uganda; musimbifredshingi@gmail.com; 2 Biosciences eastern and central Africa-International Livestock Research Institute (BecA–ILRI) Hub, P.O. Box 30709-00100, Nairobi 00100, Kenya; mutiga@uark.edu (S.M.); akellosandy@gmail.com (S.A.); F.Nganga@cgiar.org (F.N.); l.kago@cgiar.org (L.K.); m.kyalo@cgiar.org (M.K.); jjharvey@ksu.edu (J.H.); s.ghimire@cgiar.org (S.G.); 3 Department of Plant Pathology, University of Arkansas, Fayetteville, NC 72701, USA; 4 Feed the Future Innovation Lab for the Reduction of Post-Harvest Loss, and Department of Plant Pathology; Kansas State University, Manhattan, KS 66506, USA

**Keywords:** Fish feeds, Regulations, AFB_1_, Fumonisin, Uganda

## Abstract

The emergence of commercial fish farming has stimulated the establishment of fish feed factories in Uganda. However, no information is available on the safety of the feed, mainly due to lack of mycotoxin testing facilities and weak regulatory systems. A study was carried out to examine fungal colonization and mycotoxin contamination in fish feed samples (n = 147) of different types collected from nine fish farms (n = 81) and seven fish feed factories (n = 66) in the Lake Victoria Basin (LVB). Fungi were isolated in potato dextrose agar, grouped into morphotypes and representative isolates from each morphotype were identified based on the internal transcribed spacer (ITS) region of ribosomal DNA sequences. Aflatoxin B_1_ (AFB_1_) and total fumonisin (combinations of B_1_, B_2_ and B_3_; hereinafter named fumonisin) levels in feed samples were determined by enzyme-linked immunosorbent assay (ELISA). A wide range of fungi, including toxigenic *Aspergillus flavus* and *Fusarium verticillioides,* were isolated from the fish feed samples. AFB_1_ was detected in 48% of the factory samples and in 63% of the farm samples, with toxin levels <40 and >400 µg/kg, respectively. Similarly, 31% of the factory samples and 29% of the farm samples had fumonisin contamination ranging between 0.1 and 4.06 mg/kg. Pellets and powder had higher mycotoxin contamination compared to other commercially available fish feed types. This study shows AFB_1_ as a potential fish feed safety issue in the LVB and suggests a need for more research on mycotoxin residues in fish fillets.

## 1. Introduction

Uganda’s aquaculture industry is rapidly growing, with over 50% of farmed fish produced in the Lake Victoria Basin (LVB) [1]. This rapidly growing industry has led to an increase in the number of fish feed factories in Uganda. These factories’ annual production is currently estimated at 75,000 tons; a small fraction of the 120 million tons required to sufficiently supply Uganda’s aquaculture production, currently estimated at 111,023 tons per annum [2]. Fish feeds are prepared from maize bran, wheat bran, rice bran, soy meal, cotton seed cake, fish meal, bone meal and termites [3,4,5]. Unfortunately, some of these ingredients are vulnerable to contamination and colonization by fungi that produce toxic secondary metabolites called mycotoxins (e.g. aflatoxins, mainly produced by *Aspergillus flavus* and *A. parasiticus*, and fumonisins, mainly produced by *Fusarium verticillioides*) [6]. If ingested, these mycotoxins can affect the health of fish [7] and humans [8]. However, the mycotoxin levels and safety of fish feed in Uganda has not been well documented [9,10].

Feed manufacturers obtain plant-based ingredients from farmers and local markets, while the animal-based ingredients are obtained from fish landing sites and abattoirs. The ingredients are sourced from different agro-ecological zones within central, mid-western and the eastern regions of Uganda [11]. These areas are characterized by hot and humid conditions [12], which favor the growth of most mycotoxigenic fungi [13]. At the time of this study, there was limited information on fungal contaminants of fish feed in Uganda. However, mycotoxin contamination of livestock, pet and poultry feed in Uganda has been well documented [14].

Ingestion of aflatoxin-contaminated feed is known to be detrimental to fish and livestock, due to effects such as immune system suppression and growth impairment [15]. AFB_1_ is the most prevalent, potent and toxic metabolite to humans, animals and aquatic organisms [16,17]. Studies [18] have shown that the consumption of AFB_1_-contaminated feed can cause liver tumors in trout [19], impairs the growth and survival of Rohu fish (*Labeo rohita*) [20] and negatively affects weight gain, feed efficiency, serum lysozyme concentration and whole-body lipid levels in Red drum (*Sciaenops ocellatus*) [21]. Exposure of fish to fumonisin leads to a reduction in hematocrit and white blood cells, and a decline in body weight [22]. Aflatoxins and fumonisins are known human carcinogens and could be fatal if ingested in large quantities [23].

In spite of the increased number of fish farmers, there has been an increasing concern regarding low aquaculture production in the LVB [24]. Most fish feed ingredients are made from commodities (e.g., cereals) which are known to be vulnerable to mycotoxins, particularly aflatoxin, deoxynivalenol and fumonisin [25]. While mycotoxins are generally known to cause a decline in fish production, there is lack of data on AFB_1_ and fumonisin contamination levels in fish feeds and the subsequent impacts on farmed fish in the LVB. Furthermore, AFB_1_ and fumonisin are known human carcinogens, and the use of contaminated fish feeds could lead to a spillover effect of these toxins to humans [26]. Therefore, practices that can prevent mycotoxin contamination in fish feeds are important. Fish feed safety is an indicator of good quality fish and could promote fish trade, increase incomes and hence improve livelihoods of fish farmers. Mycotoxin contamination could be prevented through government policy and regulation.

The concept of mycotoxin regulation is at its infancy in many developing countries, including Uganda, due to limited capabilities and a lack of facilities for mycotoxin detection and analysis. Therefore, the regulatory authority in Uganda has adopted the limits set by other East African countries, particularly for maize and peanuts [9,27,28]. Moreover, there are no permissible limits specified for AFB_1_ and fumonisins levels in fish feed [29]. The lack of regulatory limits coupled with inadequate policy to restrain mycotoxins in fish feed have compromised fish feed safety in Uganda.

Mycotoxin regulations vary greatly by commodities and countries and may have significant implications on trade. The International Food Standards (IFS) and the European Union (EU) provide guidelines on the legal limit of mycotoxins in food and feeds [30]. The US Food and Drug Authority (FDA) has set the legal limit for AFB_1_ in animal feeds at 20 µg/kg; the World Health Organization (WHO) has 5 µg/kg as the limit, while the East African Community (EAC) standards are set at 10 µg/kg [31]. Similarly, the EU, FDA, and the EAC have set the maximum allowable fumonisin levels in animal feed at 10 mg/kg, 20 mg/kg and 10 mg/kg, respectively [32,33]. Uganda has aflatoxin limits in human food set at 10 µg/kg [9]. Despite the set standards for EAC, the lack of information on mycotoxin levels in fish feed and lack of enforcement of set standards poses a potential food safety risk to farmed fish and its consumers.

The risk of mycotoxin contamination in fish feeds can be established by investigating the profiles of mycotoxigenic fungi and the associated mycotoxins in the feed. While previous efforts have relied on morphological characterization of fungi [34], recent methods have employed molecular approaches targeting the conserved regions in the internal transcribed spacer sequences (ITS) of the ribosomal DNA gene [35]. Morphological characterization is often subjective, making identification at the species level difficult, since morphological differences can be caused by simple mutations and changes in growth media [34,35,36,37,38]. On the other hand, the molecular approach is rapid and informative in identifying multiple fungal species present in fish feed, including those with cryptic associations. Detection of mycotoxins can be performed through various methods, including enzyme-linked immunosorbent assay (ELISA), immunocapture fluorometric assay, thin-layer chromatography (TLC), and high-performance liquid chromatography (HPLC). ELISA is a quick and a relatively cheap analytical method, which has been previously used in the rapid screening of mycotoxin contamination in different commodities [31,39]. Mycotoxin testing provides information on whether feed or its ingredients are contaminated or not, and if they are, testing further informs whether the contamination levels are above or within the tolerated levels as regards the set regulations.

In this study, culturable fungi were isolated, characterized and identified from fish feed obtained from different fish farms and fish feed factories around LVB. Similarly, AFB_1_ and fumonisin were also analyzed in the same samples, with the aim of understanding fish feed safety status in the LVB. The study also examined correlations between moisture content and mycotoxins levels in feed. These findings will be valuable in the establishment and enforcement of mycotoxin standards in Uganda’s fish feed industry.

## 2. Results

### 2.1. Moisture Content in Fish Feed

For feeds from the farms, the moisture content ranged between 7.4% and 10.4%, whereas the moisture content ranged between 8.7% and 16.2% in feed from factories (Table 1). The moisture content varied significantly across farms (*p* < 0.001) and across factories (*p* < 0.001). To investigate associations between feed types and moisture content, feed samples from farms were grouped into crumbles, pellets and powder. Similarly, samples from factories were grouped into crumbles, fish meal, maize bran, pellets of four different sizes (1.5, 2.0, 3.0 and 4.0 mm), powder, and soy meal. For feed samples from farms, the moisture content was highest in pellets (10.4%) followed by that in powder (10.0%) and least in crumbles (7.4%). Moisture content in feed samples from factories was in decreasing order, as follows: pellets (4.0 mm, 16.2%; 3.0 mm, 15.4%; 1.5 mm, 15.2% and 2.0 mm, 9.7%); maize bran, 10.7%; powder, 10.2%; and fish meal, 9.6%.

### 2.2. Isolation and Identification of Fungi in Fish Feed

**Figure 1:**
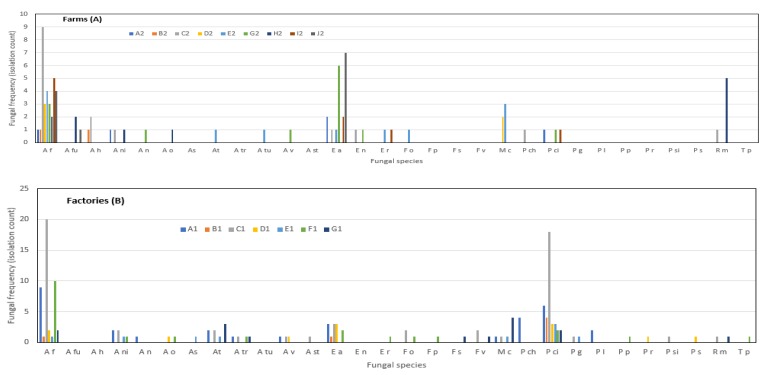
Fungal colonization frequency in fish feeds obtained from different farms (**A**) and factories (**B**) in the LVB, Uganda. Farms and factories are coded in different colours. * Species abbreviations: *A f: Aspergillus flavus, A fu: A fumigatus, A h: A hortai, A ni: A niger, A o: A oryzae, A n: A nomius, A s: A sydowi, A t: A tamari, A tr: A tritici, A tu: A tubingensis, A v: A versicolor, A st: A stellatus, E a: Eurotium amstelodami, E n: Emericella nidulans, E r: E rugulosa, F o: Fusarium oxysporum, F p: F proliferatum, F s: F sterilihyphosum, F v: F verticillioides, M c: Mucor circinelloides, P ch: Penicillium chrysogenum, P ci: P citrinum, P g: P griseofulvum, P l: P lanosum, P p: P purpurogenum, P si: P sizovae, P s: P steckii, R m: Rhizopus microsporus, *and* T p: Talaromyces purpurogenus.*

**Figure 2:**
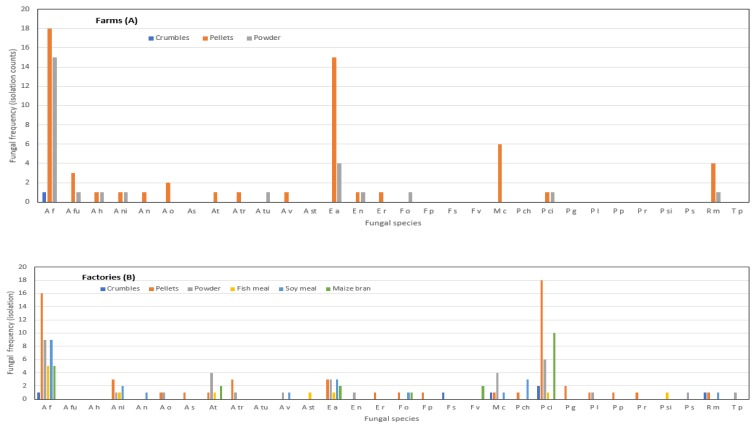
Fungal colonization frequency in different fish feed types obtained from different farms (**A**) and factories (**B**) in the Lake Victoria Basin, Uganda Feed types are coded in different colours. * Species abbreviations: *A f: Aspergillus flavus, A fu: A fumigatus, A h: A hortai, A ni: A niger, A o: A oryzae, A n: A nomius, A s: A sydowi, A t: A tamari, A tr: A tritici, A tu: A tubingensis, A v: A versicolor, A st: A stellatus, E a: Eurotium amstelodami, E n: Emericella nidulans, E r: E rugulosa, F o: Fusarium oxysporum, F p: F proliferatum, F s: F sterilihyphosum, F v: F verticillioides, M c: Mucor circinelloides, P ch: Penicillium chrysogenum, P ci: P citrinum, P g: P griseofulvum, P l: P lanosum, P p: P purpurogenum, P r: P rubens, P si: P sizovae, P s: P steckii, R m: Rhizopus microsporus, *and* T p: Talaromyces purpurogenus.*

A total of 237 fungal isolates were recovered from 147 samples, and these belonged to 30 taxa (Figure 1 and Figure 2) representing eight genera: *Aspergillus*, *Emericella*, *Eurotium*, *Fusarium*, *Mucor*, *Penicillium*, *Rhizopus* and *Talaromyces* at varying frequencies. *Aspergillus* was the most frequently occurring genera, representing 50% of the fungal population and was followed by *Penicillium* (22%). *Aspergillus flavus* (33%), *Penicilium citrinum* (17%), and *Eurotium amstelodami* (13%) were the most frequently detected fungal species. *Aspergillus viridinutans*, *Aspergillus tubingensis*, *Fusarium proliferatum*, *Fusarium sterilihyphosum*, *Mucor indicus*, *Talaromyces purpurogenus* and *Talaromyces stipitatus* were detected only once in this study. The most commonly detected fungi in the feed samples from factories were *A. flavus* (30%) and *P. citrinum* (25%). In samples from the fish farms, *A. flavus* (38%) and *Eurotium amstelodami* (22%) were the most prevalent. For samples from the factories, the least common fungi were *A. nomius*, *A. sydowi*, *A. stellatus*, *Emericellum rugulosa*, *F. proliferatum*, *M. indicus* and *Rhizomucor pusillus*. *Aspergillus tamarii*, *A. nomius*, *A. tubingensis*, *A. versicolour*, *F. oxysporum* and *T. radicus* were the least prevalent in samples from the farms.

### 2.3. Mycotoxin Contamination in Fish Feed from Factories and Farms

Based on the likelihood ratio tests, factory-sourced feed differed in the frequency of aflatoxin contamination (*χ^2^* = 52.1, df = 6, *p* < 0.0001). AFB_1_ was observed in 71% (five out of seven) of the factory-sourced feed; with an overall contamination frequency of 48%. Within the five factory-sourced feed with detectable AFB_1_, the frequency of contamination ranged from 33% to 100% and the maximum ranged from 154 to 312 µg/kg. Three feeds had detectable AFB_1_ contamination in all samples. Similarly, fumonisin contamination differed among the different factory-sourced feeds (χ^2^ = 37.2, df = 6, *p* < 0.0001) and was observed in 43% (three out seven) of the feeds with an overall contamination frequency of 29%**.** Within the three factory-sourced feeds with detectable fumonisin, contamination frequency ranged from 20% to 89% and the maximum contamination ranged from 0.6 to 2.9 mg/kg (Table 2). No correlation (*r* = 0.04, *p* > 0.05) was observed between the AFB_1_ and fumonisin levels in the feed samples from factories.

The likelihood of AFB_1_ contamination frequency differed significantly (χ^2^ = 49.9, df = 8, *p* < 0.0001) among the different farm-sourced feeds. AFB_1_ was observed in 89% (8 out of 9) of the farm-sourced feeds with an overall contamination frequency of 63%. Except for one farm-feed which had 25% contamination frequency, the rest had detectable AFB_1_ in at least 63% of their samples. One farm-sourced feed had detectable AFB_1_ in all samples. The maximum AFB_1_ contamination ranged between 97 and 403 µg/kg. Similarly, the likelihood of fumonisin contamination was significantly different among farm-feeds (χ^2^ = 45.4, df = 8, *p* < 0.0001) and was observed in 67% (six out of nine) of the feeds. Three of the farm-sourced feeds had detectable fumonisin in at least half of the sampled feed. One farm-feed had detectable fumonisin in all sampled feed. Maximum fumonisin contamination ranged from 0.1 to 4.1 mg/kg (Table 2). No correlation (*r* = −0.134, *p* > 0.05) was observed between the aflatoxin and fumonisin levels in the feed from the farms.

### 2.4. Mycotoxin Contamination in Different Feed Types

Based on analysis of means of proportions, AFB_1_ contamination differed among the feed types in the factories (n = 66, df = 5, χ^2^ = 35.5, *p* < 0.0001). Fish meal, soy meal and maize bran did not have detectable AFB_1_, while the rest of the feeds had a mean contamination ranging between 107 and 199 µg/kg. All crumbled samples were contaminated, while over 50% of pellets and powdered samples were contaminated with AFB_1_ greater than the detection limit. The proportion of AFB_1_ contamination in powdered feeds (75%) was significantly higher than the group average proportion of contamination (49%). Similarly, the proportion of fumonisin contamination differed among feed types (n = 66, df = 5, χ^2^ = 18.2, *p* = 0.0027). Fumonisin contamination was observed in two feed types, with a higher proportion of contamination in maize bran (83%) than in powdered meals (28%) (Table 3). Except for maize bran, the proportion of contamination in the rest of the feed types did not differ significantly from the group proportion mean (28%).

The proportion of AFB_1_ contamination differed significantly among the feed types in the farms (n = 81, *df* = 2, χ^2^ = 6.3, *p* = 0.04). Crumbled feed was not contaminated by any of the two mycotoxins. The percentage of samples with AFB_1_ contamination in pellets and powder form was ≥61% (Table 3).

### 2.5. Relationship between Fungal Incidence, Moisture and Mycotoxin Content in Fish Feed

Fungal frequency was not statistically correlated with mycotoxin contamination at the factories and farms. For example, factories and farms which had the highest AFB_1_ contamination frequency did not have the highest colonization frequency of the AFB_1_-producing fungi. Similarly, factories with feed that presented with the highest fumonisin frequency, had samples colonized by minor fumonisin-producing fungi, *F. oxysporum* and *F. proliferatum*. On the other hand, farms that had all samples contaminated with fumonisin did not have detectable associated fumonisin-producing fungi. Interestingly, sample colonization by minor fumonisin-producing fungi was associated with low frequencies of fumonisin contamination at the farms.

The occurrence of mycotoxin-producing fungi in specific feed types was not well-associated with frequency of contamination in factories and farms. The mean of samples with detectable AFB_1_ and the frequency of colonization by *A. flavus* were highest in pellets. Similarly, high *F. verticillioides* occurrence in maize bran was well-correlated with high frequency of fumonisin contamination at the factory. On the contrary, although crumbles had the highest frequency of AFB_1_ contamination, they had the least frequency of colonization by *A. flavus* in the factories. Furthermore, four different *Aspergillus* spp. including *A. nomius* were observed in soy meal samples, but no AFB_1_ contamination was observed in all soy meal samples from the factories. Similarly, there was no significant association observed between moisture content, mycotoxins and occurrence of fungi (*p* > 0.05) for samples collected from farms and factories.

## 3. Discussion

The current study investigated the contemporary fungal contamination of fish feed from factories and farms in the LVB of Uganda. The study site (LVB) is among the leading farmed fish producing and fish consuming regions in East Africa [10,40,41], and thus provided an excellent place to get a snapshot of potential mycotoxin problems within the aquaculture industry. The study provides information about the extent of AFB_1_ and fumonisin contamination in fish feeds. Variations in the prevalence of the two major mycotoxins at the factories and farms are shown, implying that fish feed handling and storage practices could play a key role in management of the problem. This is the first comprehensive study on the occurrence of the AFB_1_ and fumonisin in fish feed in Uganda. While the scope of the study is limited to the LVB, where these samples were obtained, some of the key drivers of mycotoxin contamination could be similar in other locations, and hence the findings are a key contribution towards reducing farmed fish exposure to these harmful toxins.

A wide diversity in feed types was observed in feed from the factories. This difference might have been caused by the several feed types produced by the factories to meet different customer preferences and the different raw materials used in feed formulation. Although some of the feed types currently present in the farms may not have been sampled in this study, we presumed that those sampled were the most popular in the region. The observed contamination levels are therefore expected to provide a guide within the geographical scope of this study. Analysis of the samples show that the average moisture content in the feed from the factories was higher than that of farm-sourced feed. This suggests that feed dried further in farmers’ storage sheds. High moisture content in feed has been associated with increased colonization of food and feedstuff by molds [31,42]. Factories have a responsibility to ensure that feeds are produced and handled under conditions which discourage fungal colonization and mycotoxin contamination. It is therefore imperative that feed manufacturers should dry the ingredients to a moisture content that reduces fungal growth, preferably at moisture content below 14% [9,43,44]; a range of drying technologies are available, which can be adapted for use by feed producers in Uganda.

This study revealed that feeds were colonized by a wide range of fungal species, some of which were mycotoxigenic. With regard to the two toxins analyzed in the current study; the known AFB_1_ producing fungi *A. flavus* and *A. nomius* were observed, but *A. parasiticus* was not. For fumonisin, the major producer, *F. verticilliodes*, as well as minor producers such as *F. oxysporum* and *F. proliferatum* were observed. Interestingly, samples in which only minor fumonisin-producing fungi were isolated had detectable fumonisin. This suggests that the ingredients used could have been contaminated prior to feed production, thus the nature of microbial species cultured from individual feeds were indicative of frequent colonizers of specific ingredients [42,45,46]. Depending on the conditions in which the ingredients were stored, even the minor toxigenic species could produce significant amounts of toxins [47]. To mitigate colonization and contamination by toxigenic fungal species, there is a need to store feed at conditions that do not favor growth and toxin production in factories and at farm sheds [48]. Several non-mycotoxigenic fungal species were observed (Figure 1 and Figure 2). There is a need to investigate whether some of the fungal species identified in this study could provide some protection against toxigenic species, given that some samples, though colonized by fungi, did not show high levels of mycotoxin contamination. Such information would be applied as a grain guard bio-control strategy.

Mycotoxin occurrence in fish feed was not always correlated with the presence of the fungus that produces it. This observation could be due to the technical challenges of culturing some species which might have been already killed by some feed preparation methods, or due to some fungi being out-competed by others in the in vitro culture environment. While such a problem could be overcome by direct PCR, such a method could face difficulties in amplifying fungal DNA due to the presence of PCR inhibitors. Although the efficacy of PCR diagnostics could have been improved through the use of previously described anti-PCR inhibitors, these methods were not used in the current study [49]. Mycotoxin contamination levels observed in the current study are a very important piece of information to all stakeholders in the aquaculture industry of Uganda and other neighboring countries. There is a need for the Ugandan government to deploy rapid diagnostics to conduct regular surveillance of mycotoxin contamination in fish feed.

For some feed types in some farms and factors, all samples had detectable AFB_1_. In addition, some samples had high AFB_1_ contamination levels with values substantially above the 5 µg/kg limit set for animal feed in Tanzania [50], in the EAC [29]; and the 20 µg/kg limit set for fish feed by Colombian regulations, FDA guidelines, European Food Safety Authority (ESFA), Codex Alimentarius and the European Commission (EC) [51]. The existence of these contaminants in the feed could pose a human food safety problem if there is significant mycotoxin carryover effect in fish tissue [26], in addition to leading to low production in the aquaculture industry. Additionally, mycotoxin contamination could compromise product quality; hence its acceptance in the regional and international market [30], which shows urgency in establishing mycotoxin regulations for fish feed. It was however noted that all fumonisin contamination levels observed in the sampled feed were within the legal limits set by the EU, FDA, and the EAC for animal feeds [32,33]. These observations show that fumonisin contamination may not be a major threat to the fish feed industry in the LVB.

Mycoflora (Figure 1 and Figure 2) and mycotoxin levels (Table 2 and Table 3) observed in this study compare well with levels observed elsewhere. For example, investigations done to assess mycoflora and mycotoxins in finished fish feed and feed ingredients in East Africa [25] revealed *Aspergillus flavus* as the most prevalent fungal species at 55% and detected DON, aflatoxin and fumonisin as the most prevalent myco-contaminants in finished fish feed and feed ingredients at 93%, 64% and 57%, respectively. Similarly, research aimed at screening for aflatoxin B1 and mycoflora related to raw materials and finished feed destined for fish in Brazil [52] revealed *Aspergillus flavus* and *P. citrinum* (70% and 75% respectively) as the most prevalent species and with all raw materials and finished products contaminated with AFB1. In the same way, investigations of multi-mycotoxin contaminations in fish feeds from different agro-ecological zones in Nigeria [49] detected fumonisin B1 as the highest contaminant (at 6.097 mg/kg). All samples analyzed were contaminated with various mycotoxins, which were produced by *Aspergillus*, *Penicillium* and *Fusarium* molds. However, studies to assess mycotoxigenic fungi and the natural co-occurrence of mycotoxins in rainbow trout (*Oncohynchus mykiss*) feed in Argentina [53], established *Eurotium* (25%) and *Pencillium* (21%) as the most prevalent species, and detected T-2toxin (64%, median 70 µg/kg) and Zearalenone (50%, median 88 µg/kg) as the most prevalent toxins. Occurrence of such mycotoxigenic fungi and mycotoxin in fish feed are a potential danger to farmed fish. This is evidenced by scenarios where mycotoxin residues have caused physiological effects on farmed fish [51], and with possible spillovers to human consumers. This has been followed by research on the possible occurrence of mycotoxin in fish muscle in China [54] which has revealed traces of ochratoxin A, zearalenone and aflatoxin B_2_ in fish muscle. Regarding these observations, aflatoxin levels detected in some fish feed samples from LVB may pose a potential risk to fish and human health, but there is a need to carry out more research to determine the gravity of this risk.

Although we did not find a significant association between moisture content and levels of contamination in this study, the observed contamination trends suggest that the physical properties of certain feed types could favor moisture retention, which favors colonization of the substrate by mycotoxigenic fungi. Fumonisin, for example, is produced in substrates with relatively higher water activity than that required for aflatoxin production [55]. Analysis was done on finished fish feed collected from fish feed factories and fish farms in form of powder, crumbles and pellets; plus, feed ingredients such as soy meal, fish meal and maize meal collected from fish feed factories. Interestingly, crumbles had relatively low moisture content but had a high AFB_1_ contamination. Low colonization of crumbles by toxigenic fungi could be attributed to feed processing conditions that might have destroyed the fungi, which had produced the toxin in the feed ingredients or to the feed storage conditions that did not favor the growth of mycotoxigenic fungi. This implies that ingredient health is very important in enhancing the safety and quality of fish feed [56,57,58].

Soymeal was colonized by *A. flavus*, but not contaminated with AFB_1_. This observation is in agreement with other studies which showed little or no AFB_1_ contamination in soybean [25,56]. Interestingly, soymeal had relatively low moisture content compared to the other feed types analyzed in this study. Feed production processes could have affected both the moisture content and fungal colonization in soymeal. Given that soymeal was colonized by *A. flavus*, it is possible that soy is not a conducive substrate for the production of AFB_1_ [59]. Mechanisms through which AFB_1_ contamination inhibition could occur include growth inhibition, chemical inhibition of biosynthetic compounds, and/or detoxification [60]. There is need to investigate the actual mechanisms through which mycotoxin contamination is inhibited in soy. If soybean contains compounds which inhibit AFB_1_ and fumonisin contamination, its meal would be a key/must have ingredient in fish and animal feeds as this would not only reduce mycotoxin exposure but also enhance the nutritional value of the feeds.

## 4. Conclusions

Mycotoxygenic fungi of genera *Aspergillus, Emericella, Eurotium, Fusarium, Mucor, Penicillum, Rhizopus,* and *Talaromyces* were isolated from commercial fish feed samples from the LVB at variable frequencies. Both AFB_1_ and fumonisin were detected in these feed samples at different concentrations. The presence of AFB_1_ and fumonisin in the feed samples did not correlate with the occurrence of mycotoxigenic fungi and moisture content. Although soybean was colonized by *Aspergillus*, it was not necessarily contaminated with AFB_1_. This study suggests that fish feed is prone to mycotoxigenic fungi and mycotoxin contamination. Appropriate precautions should be undertaken to protect fish feed from contamination. Future studies on this subject need to be expanded to other fish farming regions.

## 5. Materials and Methods

### 5.1. Study Sites and Sample Collection

**Figure 3:**
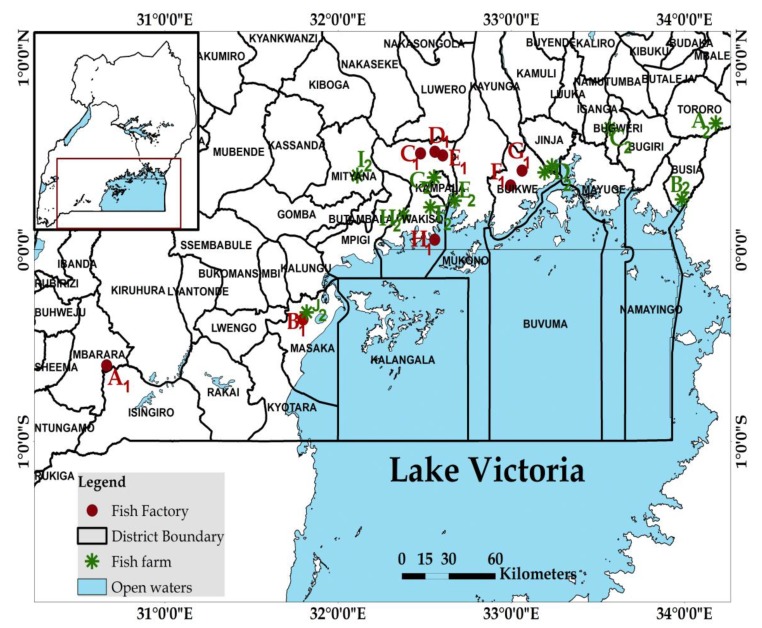
Fish farm and fish feed factory sample collection sites (Generated using ArcGIS version 10.3).

The study was carried out in the Lake Victoria Basin, Uganda (Figure 3), between May and August 2017. The region is located between E030.66336° and 033.48844°; S00.60329° and N00.61939°. It is characterized by high temperatures (24.3–29.7 °C), high annual rainfall (1000-1500.mm), at an altitude of 1034-1412 m above sea level. The region has about 5000 recorded commercial fish farmers, and eight fish processing factories.

### 5.2. Sampling Procedure

Sampling from both the farms and factories was conducted in selected districts with high frequency of aquaculture farms in LVB. Fish feed samples (n = 81) were collected from fish farms (n = 9) located in different districts in the LVB, Uganda (Figure 3). Only farms with the capacity to grow fish throughout the year using commercial feed were selected for this study. Two to five feed categories were considered at each farm and three samples were collected from each category. Samples were picked from different feed batches and locations (top, middle and bottom) in the different 50 kg sacks, since mycotoxin contamination greatly varies with space. Additional samples (n = 66) were collected from fish feed factories (n = 7), (Figure 3) following procedures like those used on the farms. Only factories manufacturing feed commercially were selected for this study. The feed samples consisted of the following categories: starters (powder and crumbles), growers (1.5 and 2.0 mm pellets), finishers (3 and 4mm pellets), and ingredients (maize bran, fish and soy meal)**.** Each sample consisted of 200-500 g of each feed type and category. In the laboratory, a subsample consisting of ⅓ of each sample was ground and used for mycotoxin analysis.

### 5.3. Fungal Isolation and Molecular Characterization

Ground samples (5 g) were diluted (10^−1^ and 10^−2^) in 50 mL of sterile distilled water, the slurry shaken at 300 rpm for 15 min at 24 °C, plated on sterile potato dextrose agar (PDA) amended with ampicillin (100 mg/kg), chloramphenicol (50 mg/kg) and streptomycin (50 mg/kg) and incubated at 28 °C for 5 days [61]. Pure cultures of different fungal species were isolated and morphologically characterized following established procedures [62].

### 5.4. Identification of Fungi using PCR-Sequencing Method

#### 5.4.1. Extraction of Fungal DNA

Direct plating of the feed followed by sub-culturing techniques was used to isolate pure cultures of different fungal species. Each fungal isolate was grown in PDA for 7–10 days. The fungus along with agar was excised from 0.25 mm^2^ (0.5 mm × 5 mm) and transferred to a sample tube. The sample was incubated overnight, lyophilized and freeze-dried at −80 °C in liquid nitrogen prior to homogenization using a Geno-Grinder (SPEX SamplePreP 2000, Canada) [63]. Fungal DNA was extracted using MagAttract 96-plant DNA extraction kit (QIAGEN, Germantown, MD, USA) following the manufacturer’s instructions. DNA was quantified using a Nanodrop spectrophotometer [64].

#### 5.4.2. PCR and Sequencing of the ITS Region

Fungal specific ITS primer pairs: ITS1F and ITS4 that amplify 18S rRNA gene (partial), ITS1, 5.8S rRNA gene, ITS2 and partial 28S rRNA gene (partial) were used in this study [65]. PCR amplification was carried out using the Bioneer Kit, with 20 µL reaction volumes containing 0.4 µL of 10 µm ITS1F, 0.4 µL of 10 µm ITS4, 16.2 µL of DDH_2_O and 3 µL of DNA template. Thermo-cycler settings were as follows: 94 °C for 3 min, thermo-cycling for 35 cycles, denaturation at 94 °C for 45 s, annealing at 48 °C for 45 s, followed by elongation at 72 °C for 45 s, and final extension at 72 °C for 10 min. For gel-electrophoresis, 3 µL of PCR product was loaded into 1.5% agarose gel that was stained with 2.5 µL of GelRed, and electrophoresis was performed at 100 V for 45 min. A 100-bp ladder was used as a size standard. The agarose gel was photographed under UV light) using an Alpha imager (Cell Biosciences Inc., Santa Clara, USA). Amplified PCR products were purified using a Qiagen PCR amplification kit and Sanger sequenced at Macrogen Sequencing Center, Netherlands. Raw sequences were analysed in CLC Main Workbench v. 7.7 [66]. Consensus sequences were searched using NCBI-BLASTn tool (https://www.ncbi.nlm.nih.gov/Blast.cgi?PAGE_TYPEBlastSearch) for homology search and fungal species identification at 99%-100% level of identity.

### 5.5. Analysis of Aflatoxin B_1_ and Fumonisin

#### 5.5.1. Sample Preparation and Mycotoxin Analysis

One third of each sample was thoroughly mixed and milled using a heavy-duty kitchen blender. Aflatoxin B1 was extracted from 5 g of ground subsamples of feed using 200 mL of extraction solution (80% Acetonitrile) and shaken at 250 rpm for 10 min. The mixture was then left to settle, and the supernatant collected for analysis. The analysis was performed in triplicate for each sample, using a commercially available Low matrix ELISA kit (Helica Biosystems Inc., Santa Ana, CA. USA. Cat. No. 981BAFL01LM-96), following the manufacture’s guidelines. The kit contained a 96-well plate coated with a mouse anti-aflatoxin monoclonal antibody, which was optimized for analysis of aflatoxin B_1_ in feeds and other matrices, and consisted of 96 non-coated mixing wells, 1.5 mL/vial of aflatoxin B_1_ standard at 0.0, 0.02, 0.05, 0.10, 0.20 and 0.40 ng/mL concentration in 50% methanol, aflatoxin B_1_ low matrix horse radish peroxidase (HRP) conjugate in a buffer with a preservative, proprietary sample diluent, stabilized tetramethylbenzidine (TMB) substrate, acidic stop solution and phosphate buffer solution (PBS) with 0.05% Tween 20. The lower and upper limits of the kit quantification, according to the manufacture’s guide were 1 and 20 µg/kg, respectively. However, samples with aflatoxin levels above the upper limit were further diluted and retested.

Fumonisin was extracted from 5 g of ground subsample with 25 mL of extraction solution (90% methanol) in 50 mL falcon tubes and shaken at a speed of 250 rpm for 10 min. Analysis was performed in triplicate for each sample, using a commercial ELISA kit (Helica Biosystems Inc., Santa Ana, CA, USA, cat No. l), following the manufacturer’s instructions. The kit contained 96 non-coated wells, 96 wells coated with a mouse anti-fumonisin monoclonal antibody which cross-reacts with three fumonisin types (B1, B2 and B3), 1.5 mL/vial of fumonisin standards at 2.5, 7.5, 20.0, 50.0 and 150.0 ng/mL concentrations, two binary fumonisin HRP-conjugates in buffer with preservative, stabilized tetramethylbenzidine (TMB), acidic solution and PBS with 0.05% Tween 20. The lower and upper limits of quantification of the kit were 0.1 and 6.0 mg/kg, respectively, based on the manufacturer’s information.

The optical density of assay contents for the two mycotoxins was determined using a micro-plate reader (BioTek Instruments, Inc. Winooski, VT, USA) using the Gen5 program at an absorbance of 450 nm.

#### 5.5.2. Quality Control

A standard in-house quality control strategy was employed as previous described by [67]. Briefly, the accuracy, precision and linearity of each ELISA plate was evaluated to determine the acceptability of the data.

### 5.6. Analysis of Moisture Content

Moisture content was determined using standard oven drying procedures as detailed by AOAC [68]. The samples analyzed using the oven drying technique were obtained from farms (n = 81) and from factories (n = 66). Briefly, samples were weighed in triplicate before and after oven-heating at 106 °C for 3 h. For statistical analysis, the ratios of the sample weight differences before and after oven-drying were converted to percentage.

### 5.7. Statistical Analysis

Data analysis (descriptive and regression) was done using JMP Pro Ver 13 (SAS Institute Inc., Cary, NC 2016) [69]. Analyses of means of proportions were conducted to compare frequencies of contamination among and within farms and factories. Analysis of variance was conducted on moisture content of the feed samples, and the means of feed types (forms) were compared using Tukey’s honest significant difference (HSD) test. AFB_1_ data were highly skewed and were transformed for normality to log (µg/kg+1) prior to further analysis [70], since the AFB_1_ kit detection range was <40 and >800 µg/kg. Similarly, percentages of samples above the upper detection limit were computed. For fumonisin, samples above the two respective detection limits were also computed. As no known limits of contamination of fish feed exist in East Africa, reporting was not based on any reference regulatory limit.

## Figures and Tables

**Table 1:** Moisture content (%) in fish feed samples from farms and factories of the Lake Victoria Basin, Uganda.

Feed Sample Type	Percentage Moisture Content							
	Farms				Factories			
	Sample Size (n)	MC ± SE	Confidence Interval (95%)		Sample Size (n)	MC ± SE	Confidence Interval (95%)	
			Lower	Upper			Lower	Upper
4.0 mm pellets	-	-	-	-	3	16.2 ± 2.2 ^a, b^	11.9	20.5
3.0 mm pellets	-	-	-	-	3	15.4 ± 2.2 ^a, b^	11.1	19.7
2.0 mm pellets	-	-	-	-	3	9.7 ± 2.2 ^a, b^	5.3	13.9
1.5 mm pellets	-	-	-	-	18	15.2 ± 0.9 ^a^	13.4	16.9
Maize bran	-	-	-	-	6	10.7±1.5 ^a, b^	7.6	13.7
Powder	18	10.0 ± 0.3 ^a^	9.5	10.5	18	10.2 ± 0.9 ^b^	8.4	11.9
Fish meal	-	-	-	-	6	9.6 ± 1.5 ^a, b^	6.5	12.6
Crumbles	3	7.4 ± 0.6 ^b^	6.2	8.7	3	9.4 ± 2.2 ^a, b^	5	13.7
Soy meal	-	-	-	-	6	8.7 ± 1.5 ^b^	5.6	11.7
Pellets	60	10.4 ± 0.1 ^a^	10.2	10.7	-	-	-	-

Mean separation using Tukey’s Honest Significant Differences. Means followed by the same letter within a column does not differ significantly (α = 0.05), SE: Standard Error.

**Table 2:** Mycotoxin contamination in fish feed from farms and factories of Lake Victoria Basin, Uganda.

	Aflatoxin (µg/kg)			Fumonisin (mg/kg)		
Feed Source	Samples (%) with AFB1 ≥ 40	Mean Contamination (AFB1 ≥ 40)	Maximum AFB1	Samples (%) with Fumonisin ≥ 0.1	Mean Contamination (≥ 0.1)	Maximum Fumonisin
**Farms**						
A2	100 (9/9)	150 ± 42.9	211	44 (4/9)	0.19 ± 0.06	0.28
B2	83 (5/6)	135 ± 28.2	175	50 (3/6)	0.17 ± 0.02	0.20
C2	25 (3/12)	154 ± 6.3	163	50 (6/12)	0.18 ± 0.06	0.26
D2	100 (6/6)	374 ± 11.5	390	17(1/6)	0.10 ± 0.001	0.10
E2	0 (0/9)	ND	ND	22 (2/9)	0.20 ± 0.02	0.23
G2	75 (9/12)	200 ± 143.6	403	0 (0/12)	ND	ND
H2	78 (7/9)	70 ± 19.0	97	100 (9/9)	1.86 ± 1.55	4.06
I2	67 (6/9)	159 ± 117.3	325	0 (0/9)	ND	ND
J2	67 (6/9)	93 ± 53.4	169	0(0/9)	ND	ND
**Total**	**63 (51/81)**			**31 (25/81)**		
**Factories**						
A1	33.3 (5/15)	211 ± 6.9	221	53(8/15)	0.7 ± 1.09	2.90
B1	0 (0/6)	ND	NA	0 (0/6)	ND	ND
C1	40 (6/15)	176 ± 96.1	312	20 (3/15)	0.1 ± 0.18	0.60
D1	100 (6/6)	145 ± 74.9	251	0 (0/6)	ND	ND
E1	0 (0/9)	ND	ND	0 (0/9)	ND	ND
F1	100 (9/9)	146 ± 54.9	224	89 (8/9)	0.8 ± 0.41	1.50
G1	100 (6/6)	90 ± 45.7	154	0 (0/6)	ND	ND
**Total**	**48 (32/66)**			**29 (19/66)**		

ND, not detected = samples did not have detectable amount of mycotoxin.

**Table 3:** AFB_1_ and fumonisin contamination in fish feed types from farms and factories in the Lake Victoria Basin, Uganda.

	Aflatoxin B_1_(µg/kg)			Fumonisin (mg/kg)		
Feed Source and Types	Samples (%) with AFB_1_ ≥ 40	Mean Contamination (AFB_1_ ≥ 40)	Maximum AFB_1_	Samples (%) with Fumonisin ≥ 0.1	Mean Contamination (≥0.1)	Maximum Fumonisin
**Farms**						
Powder	61 (11/18)	129.3 ± 37.94	180.4	22(4/18)	0.184 ± 0.06	0.26
Crumbles	0 (0/3)	ND	ND	0 (0/3)	ND	ND
Pellets	67 (40/60)	177.94 ± 20.22	403.4	35 (21/60)	0.90 ± 1.19	4.06
**Total**	**63 (51/81)**			**31 (25/81)**		
**Factories**						
1.5 mm pellets	50 (9/18)	219.0 ± 64.3	311.9	17 (3/18)	0.30 ± 0.04	0.35
2.0 mm pellets	0 (0/3)	ND	ND	0 (0/3)	ND	ND
3.0 mm pellets	100 (3/3)	168.3 ± 38.98	172.6	100 (3/3)	0.76 ± 0.21	0.96
4.0 mm pellets	100 (3/3)	169.3 ± 52.2	224.4	100 (3/3)	0.89 ± 0.27	1.19
Crumbles	100 (3/3)	117.7 ± 31.59	154.1	0 (0/3)	ND	ND
Fish meal	0 (0/6)	ND	ND	0(0/3)	ND	ND
Maize bran	0 (0/6)	ND	ND	83 (5/6)	1.86 ± 1.24	2.89
Powder	78 (14/18)	107.4 ± 54.06	2.19.6	28 (5/18)	0.59 ± 0.54	1.50
Soy	0 (0/6)	ND	ND	0 (0/6)	ND	ND
**Total**	**48 (32/66)**			**29 (19/66)**		

ND, not detected = samples did not have detectable amount of mycotoxin.

## References

[1] Gidongo Z.H. (2014). A Comparative Analysis of the Competitiveness of Tilapia and Catfish Enterprises in Mbale Sub-Region, Eastern Uganda. Master’s Thesis.

[2] Kasozi N., Rutaisire J., Nandi S., Sundaray J.K. (2017). A review of Uganda and India’s fresh water aquaculture: Key practices and experiences from each country. J. Ecol. Nat. Environ..

[3] Rutaisire J., Hassan M.R., Hect T., de Silva S.S., Tacin A.G.J. (2007). Analysis of feeds and fertilizers for sustainable aquaculture development in Uganda. Study and Analysis of Feeds and Fertilizers for Sustainable Aquaculture Development.

[4] Nalwanga R., Liti D.M., Waidbacher H., Munguti J., Zollitsch W.J. (2009). Monitoring the nutritional value of feed components for aquaculture along the supply chain—An East African case study. Livest. Res. Rural Dev..

[5] Aanyu M., Carpaij C., Widmer M. (2012). Effect of diets with graded levels of inclusion of cotton and sunflower seed cake on the growth performance and feed utilization of Nile tilapia, *Oreochromis niloticus*. Livest. Res. Rural Dev..

[6] Udomkun P., Wiredu A., Nagle M., Bandyopadhyay R., Muller J., Vanlauwe B. (2017). Mycotoxins in sub-sahara Africa: Present situation, socio-economic impact, awareness, and outlook. Food Control..

[7] Matejova I., Svobodova Z., Vakula J., Mares J., Modra H. (2016). Impact of mycotoxins on aquaculture fish species: A review. J. World Aquac. Soc..

[8] Wild C.P., Gong Y.Y. (2010). Mycotoxins and human disease: A largely ignored global health issue. Carcinogenesis.

[9] Sebunya T.K., Yourtee D.M. (1990). Aflatoxigenic Aspergilli in foods and feeds in Uganda. J. Food Qual..

[10] Kaaya N.A., Warren H.L. (2005). A review of past and present research on aflatoxin in Uganda. Afr. J. Food Agric. Nutr. Dev..

[11] Kwikiriza G., Namulawa V.T., Owori-Wadunde A., Abaho I., Ondhoro C.C. (2016). Proximate nutrient composition and cost of the selected potential fish feed ingredients in Lake Victoria Basin, Uganda. Int. J. Fish. Aquat. Stud..

[12] Kabessime E., Owuor C., Barihaihi M., Kajumba T. (2015). Monitoring and Evaluating Climate Change Adoption and Disaster Risk Reduction in Uganda.

[13] Makun H.A., Dutton M.F., Njobeh P.B., Gbodi T.A., Ogbadu G.H. (2012). Aflatoxin Contamination in Foods and Feeds: A Special Focus on Africa, Trends in Vital Foods and Contro Engineering.

[14] Rama P.J., Suhas S.J., Ashish K.S., Gaurav S., Chala M.E. (2016). Contaminants and toxins in foods and feeds. Int. J. Environ. Sci. Technol..

[15] Lizarraga-Paulin E., Moreno-Martinez E., Miranda-Castro S.P. (2011). Aflatoxins and their impact on human and animal health. An Enlarged Problem, Aflatoxin–Biochemistry and Molecular Biology by Ramon G.

[16] Hussein H., Brasel J.M. (2001). Toxicity, metabolism and impact of mycotoxins on humans and animals. Toxicology.

[17] Kennedy D., Delaney A., Koren G., Goldfrank L. (1998). Mutagens, carcinogens and teratogens. Goldfrank’s Toxicologic Emergencies.

[18] Ashley L.M. (1970). Pathology of fish aflatoxins and other anti-metabolites. A Symposium on Diseases of Fishes and Shell Fishes.

[19] Dirican S. (2015). A review of the effects of aflatoxins in aquaculture. Appl. Res. J..

[20] Ruby D.S., Masood A., Fatmi A. (2013). Effect of aflatoxin contaminated feed on growth and survival of fish *Labeo rohita* (Hamilton). Curr. World Environ..

[21] Zychowski K.E., Hoffmann A.R., Ly H.J., Pohlen Z.C., Buentello A., Romoser A., Gatlin D., Phillips T.D. (2013). The effect of aflatoxin B1 on Red drum (*Sciaenops ocellatus*) and assessment of dietary supplementation of Novasil for the prevention of aflatoxicosis. Toxin.

[22] Santos G.A., Rodrigues I., Stark V., Naehrer K., Hofstetter U., Encarnacao P. Mycotoxins in aquaculture: Occurrence in feeds components and impact on animal performance. Proceedings of the Avances en Nutricion Acuicola X–Memorias del Decimo Simposio Internacional de Nutricion Acuicola.

[23] Zain M.E. (2011). Impact of mycotoxins on human and animals. J. Saudi Chem..

[24] Bolman B., Pieter van Duijn A., Rutaisire J. (2018). Review and Analysis of Small-Scale Aquaculture Production in East. Africa Part. 4. Uganda.

[25] Marijani E., Wainaina J.M., Charo-Karisa H., Nzayisenga L., Munguti J., Gnonlonfin B., Kigadye E., Okoth S. (2017). Mycoflora and mycotoxins in finished fish feed and feed ingredients from small holder farms in East Africa. Egypt. J. Aquat. Res..

[26] Michelin E.C., Massocco M.M., Godoy S.H., Baldin J.C., Yasui G.S., Lima C.G., Rottinghaus G.E., Sousa R.L., Fernandes A.M. (2016). Carry over of aflatoxins from feed to lambari fish (*Astyanax altiparanae*) tissues. Food Addit. Contam. Part A.

[27] Uganda National Bureau of Standards (2015). Uganda Standards Catalogue as at 30 June. http://www.businesslicences.go.ug/uploads/Uganda/standards/catalogue.

[28] Okoth S. (2016). Improving the Evidence Base on Aflatoxin Contamination and Exposure in Africa: Strengthening the Agriculture–Nutrient Nexus.

[29] Grace D., Lindahl J., Atherstone C., Kangethe E., Nelson F., Wesonga T., Manyong V. (2015). Building an Aflatoxin Safe East African Community.

[30] Anukul N., Vangnai K., Mahakarnchanakul W. (2013). Significance of regulation limits in mycotoxin contamination in Asia and risk management programs at the national level. J. Food Drug Anal..

[31] Mutiga S., Were V., Hoffmann V., Harvey J., Milgroom M., Nelson R. (2014). Extent and drivers of mycotoxin contamination: Inferences from a survey of Kenya Maize mills. Phytopathology.

[32] Mazumder P.M., Sasmal D. (2001). Mycotoxins–Limits and Regulations. Ancient Sci. Life.

[33] Pinotti L., Ottoboni M., Giromini C., Dell’Orto V., Cheli F. (2016). Mycotoxin contamination in the EU feed supply chain: A focus on cereal by products. Toxins.

[34] Sharma K., Singh U.S. (2014). Cultural and morphological characterization of rhizosoheric isolates of fungal antagonist Trichodema. J. Nat. Appl. Sci..

[35] Stielow B., Bratek Z., Orezan A.K., Rudnoy S., Hensel G. (2011). Species delimitation in taxonomically difficult fungi: The case of hymenogaster. PLoS ONE.

[36] Cai L., Giraud T., Zhang N., Begerow D., Cai G., Shivas R.G. (2011). The evaluation of species concepts and species recognition criteria in plant pathogenic fungi. Fungal Divers..

[37] Balajee S.A., Borman A.M., Brandt M.E., Cano J., Cuenca-Estrella M., Dannaoui E., Guarro J., Haasa G., Kibbler C.C., Meyer W. (2009). Sequence-based identification of *Aspergilli*, *Fusarium*, and *Mucorales* species in the clinical mycology laboratory: Where are we and where should we go from here?. J. Clin. Microbiol..

[38] Toledo V., Simurro M.E., Balatti P.A. (2013). Morphological and molecular characterization of fungus, *Hirsutella* sp. isolated from planthopper and psocids in Argentina Andrea. J. Insect Sci..

[39] Nishimwe K., Bowers E., Ayabagabo J.D., Habimana R., Mutiga S.K., Maier D. (2019). Assessment of Aflatoxin and Fumonisin Contamination and Associated Risk Factors in Feed and Feed Ingredients in Rwanda. Toxins.

[40] Consulting L. (2016). Report on Market Study of the Aquaculture Market in Kenya: Kenya Market-Led Aquaculture Program (KMAP).

[41] The United Republic of Tanzania Ministry of Livestock and Fisheries, Division, MLDF (2014). Fisheries Annual Statistics Report.

[42] Kaaya A., Kyamuhangire W. (2011). The effect of storage time and agroecological zone on mold incidence and aflatoxin contamination on maize from traders in Uganda. Int. J. Food Microbiol..

[43] Sahar N., Arif S., Iqbal S., Qurat U., Afzal A., Sahar A., Jahan A., Mubarik A. (2014). Moisture content and its impact on aflatoxin levels in ready-to-use red chillies. Food Addit. Contam. Part B.

[44] Ono E.Y., Sasaki E.Y., Hashimoto E.H., Hara L.N., Correa B., Itano E.N., Sugiura T., Ueno Y., Hirooka E.Y. (2002). Post-harvest storage of corn: Effect of beginning moisture content on mycoflora and fumonisin in contamination. Food Addit. Contam..

[45] Krnjaja V., Pavlovski Z., Lukic M., Skrbic Z., Stojanovic I., Bijelic Z., Mandic V. (2014). Fungal contamination and natural occurrence of Ochratoxin a (OTA) in poultry feed. Biotechnol. Anim. Husb..

[46] Sivakumar V., Singaravelu G., Sivamani P. (2014). Isolation, characterization and growth optimization of toxicogenic molds from different animal feeds in Tamilnadu. Int. J. Curr. Microbiol. Appl. Sci..

[47] Darwish W., Ikenaka Y., Nakayama S., Ishizuka M. (2014). An overview on mycotoxin contamination of foods in Africa. J. Vet. Sci..

[48] Van Egmond H., Schothorst R., Jonker M. (2007). Regulations relating to mycotoxins in food. Anal. Bioanal. Chem..

[49] Zhonghua M., Themis J.M. (2005). Advances in understanding molecular mechanisms of fungicide resistance and molecular detection of resistant genotypes in phytogenic fungi. Crop Prot..

[50] Kajuna F.F., Temba B.A., Mosha R.D. (2013). Surveillance of aflatoxin B1 contamination in chicken commercial feeds in Morogoro, Tanzania. Livest. Res. Rural Dev..

[51] Anater A., Manyes L., Meca G., Ferrer E., Luciano F.B., Pimpao C.T., Font G. (2016). Mycotoxins and their consequence in aquaculture: A review. Aquaculture.

[52] Goncalves-Nunes E.C., Gomes-Pereira M.M., Raposo-Costa A.P., da Rocha- Rosa C.A., Pereyra C.M., Calvet R.M., Alves-Marques A.L., Cardoso-Filho F., Sanches-Muratori M.C. (2015). Screening of aflatoxin B_1_ and mycobiota related to raw materials and finished feed destined for fish. Lat. Am. J. Aquat. Res..

[53] Greco M., Pardo A., Pose G. (2015). Mycotoxigenic fungi and natural co-occurrence of mycotoxins in Rainbow trout (*Oncorhynchus mykiss*) feeds. Toxins.

[54] Wenshuo S., Zheng H., Dongxia N., Mengtong J., Wen S., Zhiyong Z., Desaeger S., Yong Z., Abio W. (2015). A reliable liquid chromatography-tandem mass spectrometry method for simultaneous determination of multiple mycotoxins in fish and dried sea foods. J. Chromatogr. A.

[55] Nesic K., Ivanovic S., Nesic V. (2014). Fusarial toxins: Secondary metaboloites of fusarium fungi. Rev. Environ. Contam. Toxicol..

[56] Xiaoying L., Lihong Z., Yu F., Yaxiong J., Lei S., Shanshan M., Cheng J., Qiugang M., Jianyun Z. (2014). Occurrence of mycotoxins in feed ingredients and complete feeds obtained from Beijing region of China. J. Anim. Sci..

[57] Whitlow W.M., Diaz D.E. (2009). Mycotoxins in feeds. Feed Stuffs.

[58] Sultana N., Hanif N.Q. (2009). Mycotoxin contamination in cattle feed and feed ingredients. Pak. Vet. J..

[59] Rodrigues I., Naehrer K. (2012). A three- year survey on the worldwide occurrence of mycotoxins in feed stuffs and feed. Toxins.

[60] Krishnamurthy Y.L., Shashikala J. (2006). Inhibition of aflatoxin B1 production of Aspergillus flavus, isolated from soybean seeds by certain natural plant products. Soc. Appl. Microbiol. Lett. Appl. Microbiol..

[61] Pradeep F., Began M., Palaniswamy M., Pradeep B. (2013). Influence of culture media on growth and pigment production by *Fusarium moniliforme* KUMBF1201, isolated from paddy field soil. World Appl. Sci. J..

[62] (1999). A Pictorial Guide for the Identification of Mold Fungi on Sorghum Grain.

[63] Pallez M., Paquali M., Bohn T., Hoffmann L., Beyer M. (2014). Validation of a quick PCR method suitable for direct sequencing: Identification of Fusarium fungal species and chemotypes for preventive approaches in food safety. Food Technol. Biotech..

[64] Desjardins P., Conklin D. (2010). NanoDrop microvolume quantitation of nucleic acids. J. Vis. Exp..

[65] Op De Beeck M., Lievens B., Busschaert P., Declerck S., Vangronsveld J., Colpaert J.V. (2014). Comparison and validation of some ITS primer pairs useful for fungal metabarcoding studies. PLoS ONE.

[66] Brozynska M., Furtado A., Henery R. (2014). Direct chloroplast sequencing: Comparison of sequencing platforms and analysis tool for whole chloroplast barcoding. PLoS ONE.

[67] Nishimwe K., Wanjuki I., Karangwa C., Darnell R., Harvey J. (2017). An initial characterization of aflatoxin B1 contamination of maize sold in the principal retail markets of Kigali, Rwanda. Food Control.

[68] AOAC (2007). Official Methods of Analysis.

[69] Mutiga S., Morales L., Angwenyi S., Wainaina J., Harvey J., Das B., Nelson R. (2017). Association between agronomic traits and aflatoxin accumulation in diverse maize lines grown under two soil nitrogen levels in Easter Kenya. Field Crops Res..

[70] Lamm S.H., Ferdosi H., Dissen E.K., Li J., Ahn J. (2015). A systematic review and meta-regression analysis of lung cancer risk and inorganic Arsenic in drinking water. Int. J. Environ. Res. Public Health.

